# The Mediating Effect of Specific Social Anxiety Facets on Body Checking and Avoidance

**DOI:** 10.3389/fpsyg.2018.02661

**Published:** 2019-01-08

**Authors:** Anne Kathrin Radix, Mike Rinck, Eni Sabine Becker, Tanja Legenbauer

**Affiliations:** ^1^LWL University Hospital Hamm for Child and Adolescent Psychiatry, Ruhr-University Bochum, Hamm, Germany; ^2^Behavioural Science Institute, Radboud University Nijmegen, Nijmegen, Netherlands

**Keywords:** body image disturbance, body checking, body avoidance, eating pathology, anxiety, safety behavior

## Abstract

**Objective:** Body checking (BC) and avoidance (BA) form the behavioral component of body image disturbance. High levels of BC/BA have often been documented to hold a positive and potentially reinforcing relationship with eating pathology. While some researchers hypothesize, that patients engage in BC/BA to prevent or reduce levels of anxiety, little is known about the mediating factors. Considering the great comorbidity between eating disorders (ED) and in particular social anxieties, the present study investigated whether socially relevant types of anxiety mediate the relationship between eating pathology and BC/BA.

**Methods:** 83 participants reporting an eating disorder and 323 healthy participants (14–25 years) took part in an online survey. Eating pathology was measured with the Eating Disorder Examination Questionnaire and Body Checking and Avoidance Questionnaire. Trait and social anxiety were assessed by means of the State Trait Anxiety Inventory (STAI-T), the Fear of Negative Evaluation (FNE) and the Social Appearance and Anxiety Scale (SAAS). Separate mediation analyses were carried out with eating pathology as independent variable, BC/BA as dependent variable and STAI, FNE, and SAAS as mediating variables.

**Results:** Anxieties correlated highly positive with eating pathology in both groups. SAAS mediated the relationship between ED pathology and BC/BA in participants with ED and mediated the relationship between ED pathology and BA in healthy participants. FNE mediated the relationship between eating pathology and BA for participants with eating pathology.

**Discussion:** SAAS mediated the relationship between eating pathology and BC/BA. Being afraid of bodily evaluations may represent a particular relevant fear that triggers safety behaviors.

## Introduction

Anxiety disorders often co-exist with eating disorders (ED) and are more prevalent in this patient group than in community samples (Swinbourne and Touyz, 2007). In particular, social anxieties embody approximately 20% of this overlap (Kaye et al., 2004). It has been hypothesized that part of the expressed ED psychopathology such as weighing oneself a lot or pinching one’s skin functions as safety behavior (Pallister and Waller, 2008; Waller, 2008). In a social context, this might imply the execution of body checking (BC) or avoidance (BA) as safety behaviors in order to deal with the experience or fear of bodily evaluations. To our knowledge, no study has investigated whether social anxieties mediate the relationship between eating psychopathology and BC/BA. To date, it is unclear whether BC/BA share a similar functionality or not. Hence, the present study will shed light on potential factors contributing to the vicious cycle between ED psychopathology and BC/BA and may offer support for the anxiolytic function of BC and BA.

The overevaluation of shape and weight forms one of the core features of (EDs; Fairburn et al., 2003) as represented in the diagnostic criteria for anorexia and bulimia nervosa of the Diagnostic and Statistical Manual of Mental Disorders (DSM-V; American Psychiatric Association, 2013). In particular, the perceptual and attitudinal aspects of body image disturbances (BID) have frequently been addressed in research (Cash and Brown, 1987; Thompson et al., 1999; Cash and Pruzinsky, 2002), whereas behavioral expressions such as BC and body avoidance (BA) have largely been neglected (Reas et al., 2002; Legenbauer et al., 2017). Shafran et al. (2004) were among the first to assess the underlying mechanisms of BC and BA in patients with ED. Here, BC refers to iterative actions in order to check one’s shape and weight such as frequent weighing, pinching the skin together, using the fit of clothes, examining specific body parts in the mirror or seeking for reassurance in order to check one’s shape and weight (Rosen in Garner and Garfinkel, 1997; Fairburn et al., 1999). Similarly, BA is attributed to all actions that hinder the direct confrontation with one’s shape and weight. Corresponding behavior concerns the refusal of being weighed, covering mirrors in the house or wearing baggy clothes, for example. Shafran et al. (2004) showed that more than half of the patients with an ED frequently engaged in BC or its avoidance. Moreover, patients did not solely engage in BC or BA. They rather alternated continuously between the two behavioral expressions of BID, suggesting a related underlying functionality to maintain the restraints in eating, control weight loss, reduce or prevent associated anxieties to gain weight. BC and BA are unlikely to only form a by-product of an ED, but actively contribute to its maintenance (Shafran et al., 2004; Bailey and Waller, 2017).

Indeed, BC and BA have been found to contribute to pathological eating in a non-clinical group (Haase et al., 2011; White and Warren, 2013). BC distinguished between people engaging in psychopathological eating behavior from those who did not (Haase et al., 2011). Similarly, BC and BA accounted for 34% of the variance when predicting eating pathology (White and Warren, 2013). More conclusive evidence comes from experimental study designs. Shafran et al. (2007) asked non-clinical participants to monitor and check their bodies in a mirror critically or non-critically. They demonstrated that participants felt more dissatisfied with their own bodies after checking their bodies critically compared to those checking their bodies non-critically. All this evidence taken together strengthens the existence of a meaningful relationship between ED pathology and the behavioral expression of BID. In order to understand how this relationship contributes to the etiology of disordered eating, insight into BC’s/BA’s sustentative functionality is crucial. For example, BC/BA has often been theorized to hold an anxiolytic function. Pallister and Waller (2008) plead to acknowledge part of ED’s symptomatology as safety behaviors. A safety behavior can be defined as any action that helps a person to gain control over a feared situation or prevent a feared catastrophe (Salkovskis, 1991). They argue that, e.g., checking your wrist functions as a way to maximize safety-seeking and prevent the feared anxiety response (e.g., a sudden weight gain). Hence, the safety behavior may keep anxiety at a tolerable level at short term but fosters anxiety in the long run as the non-occurrence of the inherent fear of gaining weight is rather attributed to checking one’s wrist than to the improbability of a sudden weight gain. A recent experiment run in a naturalistic setting displayed that checking one’s wrist every 15 min over an 8-h period led to a significant increase of fear to gain weight (Bailey and Waller, 2017). Likewise, refusing to be exposed to your own body may prevent someone from proving his/her most catastrophic fears and keeps anxieties at least momentarily at a tolerable level. In the long run, however, fears cannot be disconfirmed and underlying anxieties are strengthened. In line with this train of thought, Mountford et al. (2006) demonstrated that eating pathology is associated with safety beliefs such as “I think BC will make me more comfortable around other people.” This is supported by the fact that the degree of performed BC has been associated with anxiety (Bamford et al., 2014). The level of engaging in BA on the other hand, was found to be independent of experienced anxiety (Bamford et al., 2014). Hence, it is unclear whether both, BC and BA, are used to reduce experienced anxieties or whether they inherent a different functionality.

To obtain a better understanding of the underlying functionality, Bamford et al. (2014) stressed the necessity to examine different factors mediating the relationship between ED pathology and the behavioral components of BID. For example, De Berardis et al. (2007) found that having difficulties in identifying and describing emotions is significantly associated with a higher risk for EDs and greater body dissatisfaction. Hence, patients may engage in BC as a consequence of misinterpreting emotional cues. In the light of previously mentioned theoretical assumptions and the great overlap between anxiety disorders and EDs in general, anxiety-related constructs seem to represent fruitful candidates in mediating the relationship (for an overview see: Godart et al., 2002; Kaye et al., 2004; Swinbourne and Touyz, 2007). The central role of anxiety for BID is highlighted in a recent study, in which the difference in overestimating body size in anorexia nervosa patients compared to controls did no longer reach significance after controlling for anxiety during assessment (Øverås et al., 2014). Hence, anxiety during assessment affected body image perception. This provides first evidence for a potential link between anxiety and BID. Instead of looking at general levels of experienced anxiety, others call for more in-depth analyses of the impact of specific types of anxieties on the association between ED pathology and BID (White and Warren, 2014). Considering that social anxieties form the largest group among the comorbid anxiety disorders (Swinbourne and Touyz, 2007), constructs closely related to social anxiety seem particularly relevant. Indeed Schwalberg et al. (1992) theorized already in that social evaluative anxieties may trigger concerns over eating, shape and weight and may therefore be closely related to feared outcomes and the need to engage in safety and avoidance behaviors.

Social evaluative anxieties refer to constructs such as social physique anxiety (SPA), social appearance anxieties (SAA) and fear of negative evaluation (FNE). SPA can be defined as an anxiety people experience in response to other’s evaluation of their body shape and size (Hart et al., 1989). SAA on the other hand, refers to the anxiety experienced during the evaluation of one’s overall appearance, including but not limited to body shape (Hart et al., 2008). FNE relates to the anxiety of being negatively evaluated by others (Leary, 1983) or the loss of social approval (Utschig et al., 2010). So far, SPA has been shown to partially mediate the relationship between BC cognitions and BC behavior in a non-clinical group (Haase et al., 2007). White and Warren (2014) failed to replicate this partial mediation in a sample of college women. They rather found an expanded path model in which eating pathology predicted SPA and SAA, which in turn both predicted BC behavior. This suggests a mediating role for anxiety on the relationship on eating pathology and BC. Similarly, FNE has also been theorized to trigger BC (Rieger et al., 2010). This is supported by a longitudinal study, which depicted that FNE predicted future body dissatisfaction, one component of BID (DeBoer et al., 2013). It remains unclear whether FNE can also mediate the relationship between eating pathology and BC or BA. Theories highlighting the great overlap between ED’s and anxiety disorders, as well as the safety function ascribed to BC and BA suggest a mediating role for anxiety related constructs on the relationship between ED pathology and behavioral expressions of BID. Whereas Pallister and Waller (2008) argue that eating, weight and shape related cognitions result in safety behaviors (see page 382 in Pallister and Waller, 2008), the present study will test whether social evaluative anxieties are associated with safety behaviors and mediate the relationship between ED pathology and the behavioral component of BID. In addition, no research has investigated mediational models for social evaluative anxieties on both BC and BA.

Hence, the present study aims at testing bivariate associations between SAA, FNE, trait anxiety (STAI-T), BC, BA, and ED pathology (EDEQ). We hypothesize a positive association between EDEQ and BC/BA, as well as between SAA, FNE, STAI, and BC/BA. Considering that SAA and SPA are highly correlated with each other (White and Warren, 2014) and SAA comprises the anxiety of one’s overall appearance including body shape (Hart et al., 2008), the present study focusses on the mediating influence of SAA rather than on both constructs. Subsequent mediational analyses shall answer the question to what extent overall trait anxiety, SAA or FNE mediate the relationship between ED pathology and the need to engage in BC or BA in a group of healthy participants compared to participants reporting an ED. We hypothesize that socially relevant types of anxiety (assessed by SAAS and FNE) rather than an overall level of trait anxiety (assessed by STAI-T) will exert a mediating effect on the relationship between ED pathology and BC/BA. Secondly, we hypothesize that those effects will be particularly prominent in participants reporting an ED rather than in healthy participants.

## Materials and Methods

### Ethics Statement

This study was carried out in accordance with the recommendations of the ethics committee of the medical faculty of the Ruhr-university Bochum. The protocol was approved by the ethics committee of the Ruhr-university Bochum (15-5500-BR). All subjects gave written informed consent in accordance with the Declaration of Helsinki. Parents or legal guardians were not obliged to provide written informed consent for the non-adult participants aged 14 years or older.

### Participants

Overall, 524 participants from the general population took part in the present study. All participants filled out the questionnaires online (via EvaSys Version 7.0; Electric Paper Evaluationssysteme GmbH, 2016) and could win one out of three 20 € Amazon vouchers. They were female between 14 and 25 years old. Of the initial sample (*N* = 524), 47 participants had to be excluded as they did not meet the inclusion criteria (male: *n* = 8, wrong age: *n* = 39). In addition, all participants reporting any psychiatric disorder other than an eating disorder were excluded (*n* = 71) leaving a final sample of 83 participants reporting an eating disorder (ED) and 323 participants reporting no psychiatric disorder (*N* = 406). Participants were mainly German (Participants with ED pathology: 94.0%; healthy participants: 93.2%) and spoke German as their mother tongue (Participants with ED pathology: 96.4%; healthy participants: 95.0%).

### Measures and Materials

#### Eating Disorder Pathology

The Eating Disorder Examination Questionnaire (EDE-Q; Fairburn and Beglin, 1994; German version: Hilbert et al., 2007) was used to assess the general degree of eating-disordered psychopathological behavior, as well as “restraint eating,” “eating concerns,” “weight concerns,” and “shape concerns” on four different subscales. Global eating pathology is measured by summing up all four subscales items and dividing the resulting total by four. The German version of the EDE-Q shows good convergent and discriminatory validity, high reliability and high retest reliability (Hilbert et al., 2012). The total score in the current sample showed excellent reliability with Cronbach’s α = 0.93.

#### Body Checking and Avoidance

The Body Checking and Avoidance Questionnaire (BCAQ; Legenbauer et al., 2017) is a 27-item instrument to measure eating-disorder-related psychopathological forms of BC, BA, and reassurance seeking on three subscales. Items can be answered on a 4-point Likert scale ranging from 1 (not at all true) to 4 (very true). Example items are “I make certain body movements to check whether my fat wobbles” or “I wear clothes that cover my whole body, even in the summer,” The BCAQ offers excellent internal consistency for BC and BA (Cronbachs α = 0.92), acceptable internal consistency for reassurance seeking (Cronbachs α = 0.79), as well as a good convergent and divergent validity. Excellent to good reliability was replicated in the present sample (checking: Cronbachs α = 0.93, avoidance: Cronbachs α = 0.91, reassurance: Cronbachs α = 0.80).

#### Trait Anxiety

The trait version of the State-Trait Anxiety Inventory (STAI-T; Spielberger, 1983) was employed to assess general level of an anxious temperament. It is a 20-item instrument that can be answered on a 4-point Likert scale ranging from 1 (almost never) to 4 (almost always). Example items are “I worry too much over something that really does not matter.” or “I am a steady person.” Trait anxiety is measured by summing up all items into a total score. The German version of the STAI-T (Laux et al., 1981) has high internal validity, good convergent and divergent validity, as well as good test-retest reliability. In the present sample, an excellent reliability (Cronbachs α = 0.95) was also found.

#### Fear of Negative Evaluation

The Brief Fear of Negative Evaluation (BFNE; Leary, 1983) is the short version of the original FNE Scale (Watson and Friend, 1969). It is a 12-item questionnaire that can be answered on a 5-point Likert scale ranging from 0 (not at all) to 4 (extremely). It measures the anxiety to be negatively evaluated by others. Example items are “I am afraid that others will not approve of me” or “I am usually worried about what kind of impression I make,” FNE is assessed by summing up all 12 items into a total score. The German version of the BFNE has excellent internal validity, good convergent and divergent validity, as well as high test-retest validity (Reichenberger et al., 2015). In the current sample the BFNE exhibited excellent internal consistency (Cronbachs α = 0.96).

#### Social Appearance Anxiety

The Social Appearance Anxiety Scale (SAAS; Hart et al., 2008) is a 16-item measure that assesses the anxiety of being negatively evaluated by others because of one’s overall appearance. Items can be rated on a 5-point Likert-type scale ranging from 1 (not at all) to 5 (extremely). Examples encompass items such as “I feel nervous when having my picture taken,” or “I am concerned people would not like me because of the way I look.” The degree of social appearance anxiety is measured by summing up all 16 items into one total score. The English version shows good internal validity, good test-retest reliability, as well as good factor validity (Hart et al., 2008). In the present study, a German version of the questionnaire translated into German and back translated into English by the author group and a German/English translator has been used. Any differences between the original version and the retranslation were discussed among the author group to improve the quality of the German translation. The translated questionnaire showed excellent internal consistency in the present sample (Cronbachs α = 0.97).

#### Body Mass Index

Participants’ Body Mass Index (BMI; kg/m^2^) was calculated using self-reported weight and height and used as a covariate in subsequent analyses as the present body of knowledge is still inconsistent to what extent BMI influences the association between anxiety and eating pathology.

### Statistical Analysis

Participants missing descriptive data or missing many data points were dealt with when running the analysis by means of listwise deletion. The α-criterion was set to 0.05 for all subsequent analyses. Statistics were performed with IBM^®^ SPSS^®^ Statistics version 24 (IBM Corp. Released, 2016).

Initially, independent *t*-tests were conducted to assess differences between the two groups (participants with reported ED vs. healthy participants). Subsequently, Pearson’s R was calculated for both groups (with reported ED/healthy) to assess bivariate associations between ED pathology, BC, BA, STAI-T, FNE, SAAS, and BMI. Mediation analyses were performed using a bootstrapping approach with the SPSS macro PROCESS provided by Preacher and Hayes (2008), based on 10,000 bootstrap samples and a 95% confidence interval. The bootstrapping method produces confidence intervals (CIs) to test for significance, with values not crossing zero corresponding to significance at the *p* < 0.05 level. To ensure that reporting an ED or not had a significant impact on our mediational models, it was entered as covariate in a first general mediational model including all participants. Reporting an ED diagnosis had a significant impact on the entire model for the relationship between ED pathology and BC (ß = 0.22; *t* = 2.55, *p* < 0.05, CI [0.05, 0.39]) and BA (ß = 0.18; *t* = 1.98, *p* < 0.05, CI [0.00, 0.36]). Hence, separate sets of analyses were performed for participants reporting an ED or not.

Four separate sets of indirect effect analyses were performed with eating pathology as independent variable and BC or BA as dependent variable. STAI-T, SAA, and FNE were entered as mediating variables. Two sets looked at the indirect effects for participants reporting an ED and two sets were performed for healthy participants.

The first set was run in a group of participants reporting an ED, the second set in a sample of healthy participants. In both sets, STAI-T, FNE, and SAA were used as mediating variables and the degree of BC served as dependent variable. The third set was run in a group of participants reporting an ED, the fourth set in a sample of healthy participants. In the third and fourth set, STAI-T, FNE, and SAA were again used as mediating variables and BA was entered as dependent measure.

## Results

### Demographic Information

Detailed information on participant’s age, BMI, trait anxiety, FNE, SAAS, global eating pathology, BC, and BA are presented in Table 1. Participants reporting an ED were on average a few years younger, had a lower BMI, were significantly more anxious and had a greater eating pathology compared to participants reporting no ED.

**Table 1:** Group comparisons of the two groups regarding age, body mass index, trait anxiety, fear of negative evaluation, social appearance anxiety, global eating pathology, body checking, and avoidance.

	Participants with ED (*n* = 83)		Healthy participants (*n* = 323)		Group comparisons		
Variables	*M*	*SD*	*M*	*SD*	*T*	*df*	*p*
Age (years)	20.36	3.17	21.92	2.54	4.72	404	<0.001
BMI (kg/m^2^)	19.81	3.06	22.10	4.66	4.21	400	<0.001
STAI-T	56.24	12.66	43.27	11.88	-8.75	404	<0.001
FNE	45.28	11.28	36.26	12.70	-5.90	404	<0.001
SAAS	54.10	17.97	38.10	17.02	-7.55	404	<0.001
EDEQ-Global	4.85	1.62	2.77	1.43	-11.54	404	<0.001
BC	2.78	0.88	1.83	0.69	-10.52	404	<0.001
BA	2.41	0.82	1.63	0.61	-9.59	404	<0.001


### Associations Between Eating Pathology and Anxiety

As expected STAI-T, FNE, and SAAS were strongly positively correlated with general eating pathology, as well as BC and its avoidance across both groups (see Table 2 for participants with reported ED; see Table 3 for healthy participants). BMI was only correlated with general eating pathology, avoidance behavior and social appearance anxiety for healthy participants. BMI did not correlate with eating pathology or anxiety instruments for participants with an eating disorder.

**Table 2:** Bivariate associations for participants reporting no psychiatric disorder.

Pearson R	Eating pathology	Body checking and avoidance		Trait anxiety	Specific anxieties		BMI
	EDEQ_Global	BCAQ_BC	BCAQ_AV	STAI-T	FNE	SAAS	
EDEQ_Global	1	0.733^∗∗^	0.673^∗∗^	0.587^∗∗^	0.577^∗∗^	0.729^∗∗^	0.153^∗∗^
BCAQ_Checking	0.733^∗∗^	1	0.578^∗∗^	0.522^∗∗^	0.508^∗∗^	0.600^∗∗^	0.035
BCAQ_Avoidance	0.673^∗∗^	0.578^∗∗^	1	0.522^∗∗^	0.481^∗∗^	0.672^∗∗^	0.146^∗∗^
STAI-T	0.587^∗∗^	0.522^∗∗^	0.522^∗∗^	1	0.640^∗∗^	0.700^∗∗^	-0.030
FNE	0.577^∗∗^	0.508^∗∗^	0.481^∗∗^	0.640^∗∗^	1	0.696^∗∗^	0.003
SAAS	0.729^∗∗^	0.600^∗∗^	0.672^∗∗^	0.700^∗∗^	0.696^∗∗^	1	0.133^∗^
BMI	0.153^∗∗^	0.035	0.146^∗∗^	-0.030	0.003	0.133^∗^	1


*^∗^Means *p* < 0.05, ^∗∗^means *p* < 0.01.*

**Table 3:** Bivariate associations for participants reporting an eating disorder.

Pearsons R	Eating pathology	Body checking and avoidance		Trait anxiety	Specific anxieties		BMI
	EDEQ_Global	BCAQ_BC	BCAQ_AV	STAI-T	FNE	SAAS	
EDEQ_Global	1	0.765^∗∗^	0.732^∗∗^	0.726^∗∗^	0.533^∗∗^	0.583^∗∗^	0.030
BCAQ_Checking	0.765^∗∗^	1	0.649^∗∗^	0.540^∗∗^	0.472^∗∗^	0.592^∗∗^	-0.104
BCAQ_Avoidance	0.732^∗∗^	0.649^∗∗^	1	0.639^∗∗^	0.473^∗∗^	0.621^∗∗^	0.085
STAI-T	0.726^∗∗^	0.540^∗∗^	0.639^∗∗^	1	0.680^∗∗^	0.650^∗∗^	0.022
FNE	0.533^∗∗^	0.472^∗∗^	0.473^∗∗^	0.680^∗∗^	1	0.817^∗∗^	0.125
SAAS	0.583^∗∗^	0.592^∗∗^	0.621^∗∗^	0.650^∗∗^	0.817^∗∗^	1	0.194
BMI	0.030	-0.104	0.085	0.022	0.125	0.194	1


*^∗∗^Means *p* < 0.01.*

### Mediation for Eating Pathology – BC Relationship

#### Participants Reporting an ED

**FIGURE 1:**
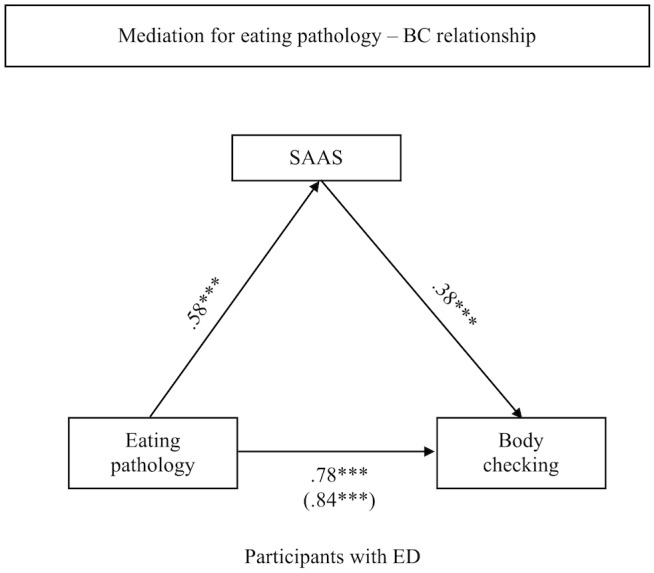
Indirect effect of SAAS on the association between eating pathology and body checking for participants reporting an ED.

Mediation was tested for STAI-T, FNE, and SAAS on the relationship between eating pathology and BC. A significant standardized indirect effect was found for SAAS (ß = 0.20, CI [0.06, 0.35]). The standardized indirect effects for STAI-T (ß = -0.10, CI [-0.28, 0.07]) and FNE (ß = -0.05, CI [-0.19, 0.08]) were non-significant. In this analysis, the standardized direct effect of eating pathology on BC was weaker but still significant (ß = 0.78; *t* = 6.96, *p* < 0.001, CI [0.56, 1.01]) compared to the total effect (ß = 0.84; *t* = 10.69, *p* < 0.001, CI [0.68, 0.99]). The results suggest a partial mediation with SAAS as significant mediator (see Figure 1).

#### Healthy Participants

Using the same approach, mediation was tested for STAI-T, FNE, and SAAS on the relationship between eating pathology and BC. No significant indirect effect was found for STAI-T (ß = 0.05, CI [-0.02, 0.12]), for FNE (ß = 0.04, CI [-0.02, 0.10]) nor for SAAS (ß = 0.04, CI [-0.07, 0.14]) as potential mediators.

### Mediation for Eating Pathology – BA Relationship

#### Participants Reporting an ED

**FIGURE 2:**
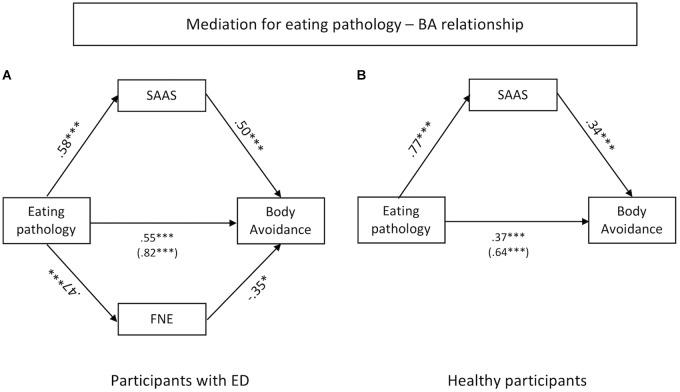
Indirect effects of SAAS and FNE on the association between eating pathology and body avoidance for participants reporting an ED **(A)** and healthy participants **(B)**.

Mediation was tested for STAI-T, FNE, and SAAS on the relationship between eating pathology and BA. A significant standardized indirect effect was found for SAAS (ß = 0.26, CI [0.11, 0.44]) and FNE (ß = -0.14, CI [-0.37, -0.01]). The standardized indirect effects for STAI-T (ß = 0.14, CI [-0.01, 0.31]) was non-significant. In this analysis, the standardized direct effect of eating pathology on BA was weaker but still significant (ß = 0.55; *t* = 4.65, *p* < 0.001, CI [0.31, 0.78]) compared to the total effect (ß = 0.82; *t* = 9.67, *p* < 0.001, CI [0.65, 0.99]). The results suggest a partial mediation with SAAS and FNE as significant mediators (see Figure 2A).

#### Healthy Participants

Again, mediation was tested for STAI-T, FNE, and SAAS on the relationship between eating pathology and BA. A significant indirect effect was found for SAAS (ß = 0.28, CI [0.15, 0.41]). No significant indirect effect was found for STAI-T (ß = 0.03, CI [-0.05, 0.12]) nor for FNE (ß = -0.03, CI [-0.09, 0.03]) as potential mediator. Also, the standardized direct effect of eating pathology on BA was weaker but still significant in this analysis (ß = 0.37; *t* = 6.75, *p* < 0.001, CI [0.26, 0.47]) compared to the total effect (ß = 0.64; *t* = 16.31, *p* < 0.001, CI [0.56, 0.71]). The results suggest a partial mediation for SAAS (see Figure 2B).

## Discussion

The present study strived to elucidate in what way SAA, FNE, and STAI-T mediate the relationship between ED pathology and the need to engage in BC or BA. Two separate sets of mediation analyses were carried out in a group of participants reporting an ED and two sets in a group of healthy participants. It was hypothesized that ED pathology is positively correlated with BC and BA. In addition, positive correlations were predicted for SAA, FNE, STAI-T, and BC/BA. Furthermore, it was expected that socially relevant types of anxiety (SAA and FNE) rather than general trait anxiety (STAI-T) mediate the relationship between ED pathology and BC/BA.

Positive associations were found for ED pathology and the degree of BC/BA, as well as for assessed levels of anxiety and BC/BA in both groups. BMI on the other hand, had no significant associations with BC/BA or measured levels of anxiety. Our hypothesis that rather socially relevant anxieties mediate the relationship between ED pathology and BC and avoidance was confirmed for participants reporting an ED. SAA was found to be a consistent mediator for the relationship between ED pathology and the behavioral component of BID measured by BC and BA in participants reporting an ED. In addition, it mediated the relationship between ED pathology and BA in healthy participants. FNE on the other hand, was only a predictive mediator for the relationship between ED pathology and BA in participants reporting an ED. STAI-T had no predictive value for the relationship between ED and BID in both groups.

### Role of Social Appearance Anxieties for Maintaining BID

The present findings highlight the critical role SAA holds in the interaction and maintenance of eating pathology and BID. The findings are in line and build upon research suggesting that BC is used to reduce distress experienced from continuous preoccupation with body size and shape (Reas et al., 2002; Mountford et al., 2006). More specifically, the present findings lend support for the assumption that people engage in BC to counteract body-related anxieties (Legenbauer et al., 2017). Depending on the strength of experienced SAA the need to engage in BC may be triggered to prevent or reduce experienced distress and body-related anxieties. Similarly, SAA mediated the relationship between eating pathology and BA, suggesting a similar underlying mechanism for BC and BA. To date, little research has investigated the underlying mechanisms of BA. It is known that patients alternate between checking and avoidance when it comes to their body shape (Shafran et al., 2004). Likewise, BA may be used as an alternative coping strategy to reduce body-related anxieties. For example, I wear baggy clothes or avoid looking into the mirror in order to avoid the confrontation with my own silhouette or negative feedback from others.

For healthy participants, SAA did also partially mediate the relationship between eating pathology and BA. Considering that we found similar associations in healthy participants and participants reporting an ED, the present findings may point toward a more general phenomenon. It is possible that avoiding a feared object independent of its specific nature and context (e.g., body, scale, spider, seeing blood, etc.) may represent a rather general strategy to cope with experienced levels of anxieties independent of a specific diagnoses or psychopathological syndrome.

### Role of Fear of Negative Evaluation for Body Avoidance

As a second core finding, FNE did partially mediate the relationship between ED pathology and BA in participants reporting an ED. Considering the great overlap and shared vulnerability between social anxiety disorders and EDs (Godart et al., 2002; Kaye et al., 2004; Swinbourne and Touyz, 2007), as well as the predictive value of FNE on future body dissatisfaction (DeBoer et al., 2013), the present findings fit well with theoretical assumptions. Simultaneously, the present findings suggest a similar functionality for BC and BA in the context of experienced anxiety. This contradicts Bamford et al. (2014) who found the level of BA being rather dependent on weight than experienced levels of anxiety in a sample of weight restored and underweight patients with anorexia nervosa. Taking into account that the present sample included participants with all kinds of EDs and did not represent an underweight sample, it is possible that the mediating impact of FNE is limited to specific weight classes or does not apply to patients with anorexia nervosa.

### Limitations

Besides the cross-sectional design, a few limitations of the present study need to be raised. First of all, data has been collected via an online survey. Hence, collected data such as reported diagnoses, weight, height, etc., could not be verified by professionals. Moreover, we did not differentiate between different diagnostic groups. In the future, it would be interesting to investigate the mediating effect of SAA and FNE on eating pathology and BC/BA across the different ED diagnostic groups. It is possible that different patient groups use diverse coping strategies to deal with their anxieties. Investigating the impact of SAA on the relationship between eating pathology and BC/BA in a larger sample across different diagnostic groups may enhance our understanding on how the behavioral component of BID is strengthened and maintained.

## Conclusion

All in all, the present findings support the idea that social anxieties and in particular SAA and FNE impact the relationship between eating pathology and the behavioral expression of BID. The present findings highlight the significant role of socially relevant types of anxiety that mediate the interaction between ED pathology and BC/BA. It may offer new insights on where to start or how to break through the vicious cycle between ED pathology and BC/BA. At the same time, it offers support for the anxiolytic function of BC and BA. As the present data is only cross-sectional, future research would largely benefit from longitudinal or experimental designs to make predictions about the temporal course or direct impact of social anxieties on the usage of BC and BA. It would be interesting to integrate other factors such as internalization of societal norms such as the “thin ideal” when looking at the mediating effect of social anxiety on BC/BA. For example, Utschig et al. (2010) proposed that participants high on FNE may be more likely to adopt societal norms such as the “thin ideal” to prevent negative social feedback. It would be an intriguing question to investigate whether the degree of believing in societal norms affects the relationship between FNE and BC/BA.

## Data Availability

The raw data supporting the conclusion of this manuscript will be made available by the authors, without undue reservation, to any qualified researcher.

## Author Contributions

All authors designed the study, planned the analyses, contributed to and have approved the final manuscript, and had full access to the study data. AR conducted the study, conducted the literature review, wrote the research summaries, and wrote the first draft of the manuscript. AR and TL analyzed the data.

## Conflict of Interest Statement

The authors declare that the research was conducted in the absence of any commercial or financial relationships that could be construed as a potential conflict of interest.
